# Proteinuria reduction as a surrogate endpoint for clinical study of IgA nephropathy in Japanese patients: data from the J-CKD-DB-Ex

**DOI:** 10.1007/s10157-025-02788-4

**Published:** 2025-11-18

**Authors:** Naoki Kashihara, Seiji Itano, Takaya Nakashima, Tadahiro Goto, Keisuke Yoshihara, Shunsuke Eguchi, Kazuma Iekushi, Yoshitaka Isaka, Hajime Nagasu, J-CKD-DB collaborators

**Affiliations:** 1 Geriatric Medical Center, Kawasaki Medical School, 577 Matsushima, Kurashiki, Okayama 701-0192 Japan; 2 Department of Nephrology and Hypertension, Kawasaki Medical School, Kurashiki, Okayama Japan; 3 Department of Anesthesiology and Intensive Care Medicine, Nagasaki University Graduate School of Biomedical Sciences, Nagasaki, Japan; 4 Department of Health Data Science, Graduate School of Data Science, Yokohama City University, Kanagawa, Japan; 5 Medical Affairs, Novartis Pharma K.K., Tokyo, Japan; 6 Department of Nephrology, Osaka University Graduate School of Medicine, Osaka, Japan

**Keywords:** IgA nephropathy, Chronic kidney disease, Surrogate endpoint

## Abstract

**Background:**

Early reduction in proteinuria has been validated as a surrogate endpoint for IgA nephropathy (IgAN) in Western trials and is used for accelerated drug approval. However, its applicability to Japanese patients remains unclear. We aimed to evaluate the association between early proteinuria reduction and long-term renal outcomes in Japanese patients with IgAN.

**Methods:**

This retrospective observational study used data from J-CKD-DB-Ex, a real-world database of CKD in Japan. Adult participants with IgAN, baseline urine protein/creatinine ratio (UPCR) ≥ 0.5 g/gCr, and eGFR ≥ 30 mL/min/1.73 m^2^ were included. The exposure was a ≥ 30% UPCR reduction at 9–12 months after the index date (UPCR reduction group), vs participants without such reduction (non-UPCR reduction group). The primary endpoint was a composite of 40% decline in eGFR from baseline or onset of CKD stage G5. Cox proportional hazards and linear mixed-effects models evaluated the association between UPCR reduction, renal events, and eGFR slope.

**Results:**

Among 385 participants (mean observation period 2,040 days), 245 achieved ≥ 30% reductions in UPCR. The UPCR reduction group showed significantly lower cumulative incidence of renal composite events than the non-UPCR reduction group. Annual eGFR decline was slower in the UPCR reduction group than that in the non-UPCR group (−1.9 vs −3.4 mL/min/1.73 m^2^/year). Greater UPCR reductions were linearly associated with more favorable eGFR slope.

**Conclusions:**

Early proteinuria reduction is associated with decreased risk of renal failure and attenuated eGFR decline in Japanese patients with IgAN, supporting its validity as a surrogate endpoint for renal prognosis.

**Supplementary Information:**

The online version contains supplementary material available at 10.1007/s10157-025-02788-4.

## Introduction

In clinical trials for kidney diseases, hard endpoints are undeniably the occurrence of end-stage kidney disease (ESKD) and kidney-related death. However, the low incidence of hard endpoints when investigating early stages of kidney disease or rare diseases presents a significant obstacle to conducting clinical trials and developing new drugs [1]. Consequently, surrogate endpoints that can predict hard endpoints early after the start of an intervention trial have been developed. For instance, doubling of serum creatinine and a 40% decrease in estimated glomerular filtration rate (eGFR) have been established as surrogate endpoints for ESKD in chronic kidney disease clinical trials [2].

IgA nephropathy (IgAN) is a common cause of chronic glomerulonephritis. The development of specific and effective therapeutic agents to reduce the number of patients initiating dialysis due to IgAN is an urgent issue. However, IgAN typically exhibits slow progression, with several cases having prolonged clinical courses [3]. Therefore, the number of patients with IgAN reaching an endpoint may be small in clinical trials for new treatments, even with existing surrogate endpoints, potentially making trial execution difficult. Consequently, the development of surrogate endpoints that can predict long-term improvement in renal prognosis early in clinical trials for IgAN is crucial.

Against this background, in the United States, the Kidney Health Initiative, a partnership between the American Society of Nephrology and the U.S. Food and Drug Administration, has validated and supported the use of proteinuria reduction as a surrogate endpoint in IgAN [4]. Accordingly, new drugs for IgAN such as budesonide (NefIgArd trial [5]), sparsentan (PROTECT trial [6]), iptacopan (APPLAUSE-IgAN trial [7]), and atrasentan (ALIGN trial [8]) have received FDA accelerated approval based on proteinuria reduction at 9 months.

Although there is a conditional approval system in Japan, it is limited to certain diseases, and IgAN is not considered in scope. Therefore, the effective drugs mentioned above are available overseas but not in Japan, causing a drug lag and disadvantaging Japanese patients. Given that the incidence, prevalence, main treatments, and prognosis of IgAN in Japanese patients differ from those in Western and other Asian countries [9], whether the surrogate endpoint recommended in the United States can be applied to Japanese patients with IgAN remains unclear. Therefore, this study aimed to investigate the external validity of applying early proteinuria reduction as a surrogate endpoint in Japanese patients with IgAN. Particularly, we examined the association between proteinuria reduction and subsequent renal failure events in patients with IgAN using J-CKD-DB-Ex, a real-world database of CKD in Japan.

## Materials and methods

### Data source and inclusion/exclusion criteria

The J-CKD-DB-Ex is an extensive, multi-institutional registry which includes data from electronic health records of patients with CKD across 21 Japanese university hospitals [10, 11]. Briefly, this database uses SS-MIX2 standardized storage to automatically extract data on CKD cases from electronic medical records. The criteria for registering participants with CKD in the J-CKD-DB-Ex are as follows: (1) age ≥ 18 years and (2) proteinuria ≥ 1 + (dipstick test) and/or eGFR of < 60 mL/min/1.73 m^2^ during the study period.

Herein, participants with IgAN, identified using ICD-10 codes, were extracted from the J-CKD-DB-Ex database between January 1, 2014, and December 31, 2022 (9-year period). The index date was defined as the first point at which the urine protein/creatinine ratio (UPCR) was calculated in the time-series laboratory values for each participant. The eGFR measurement closest to the index date within a range of ± 30 days from the index date was defined as the baseline eGFR. The inclusion criteria were as follows: (1) UPCR of ≥ 0.5 g/gCr at the index date, and (2) baseline eGFR value ≥ 30 mL/min/1.73 m^2^. The exclusion criteria were as follows: (1) patients without a UPCR measurement within 9–12 months after the index date, (2) those without an eGFR measurement during the observation period, and (3) those using immunosuppressants (excluding corticosteroids). We excluded participants receiving immunosuppressants, considering the possibility of other coexisting autoimmune diseases. Participants with concomitant diagnoses of IgA vasculitis (Henoch–Schönlein purpura) and IgAN were included.

### Exposure definition

The exposure was defined as a ≥ 30% reduction in UPCR at 9 months after the index date (UPCR reduction group), with the comparison group consisting of participants without a ≥ 30% reduction in UPCR within that period (non-UPCR reduction group) [12, 13]. Values obtained within the period of 9–12 months after the index date were permitted for the second UPCR measurement.

### Definition of outcome and observation period

The primary endpoint was a time‑to‑event composite of a 40% decline in eGFR from baseline or confirmed new onset of CKD G5 (eGFR ≤ 15 mL/min/1.73 m^2^), with the event date defined as the first occurring component. The secondary endpoints were the occurrence of a 40% decline in eGFR from baseline eGFR and new onset of CKD stage G5 (eGFR < 15 mL/min/1.73 m^2^) individually, and the inter-group difference in the annual rate of eGFR decline (eGFR slope). The observation period was set as the duration from the end of the period used to define the explanatory variable until the occurrence of the outcome or the final eGFR measurement.

### Statistical analysis

Baseline characteristics were compared between the UPCR reduction and non-reduction groups using the Wilcoxon rank-sum test for continuous variables and the chi-squared or Fisher’s exact test for categorical variables. Kaplan–Meier analysis with log-rank tests and Cox proportional hazards models were used to estimate hazard ratios (HRs) with 95% confidence intervals. Annual eGFR decline was assessed using linear mixed-effects models with all available measurements. Prespecified subgroup and sensitivity analyses were conducted. Covariates included age, sex, baseline eGFR, baseline UPCR, and prescription of renin–angiotensin system (RAS) inhibitors and corticosteroids. Further details of the statistical methods, including assumption testing, spline analyses, and model specifications, are provided in the supplementary material. Statistical significance was set at *P* < 0.05.

## Results

### Baseline characteristics

**Fig. 1:**
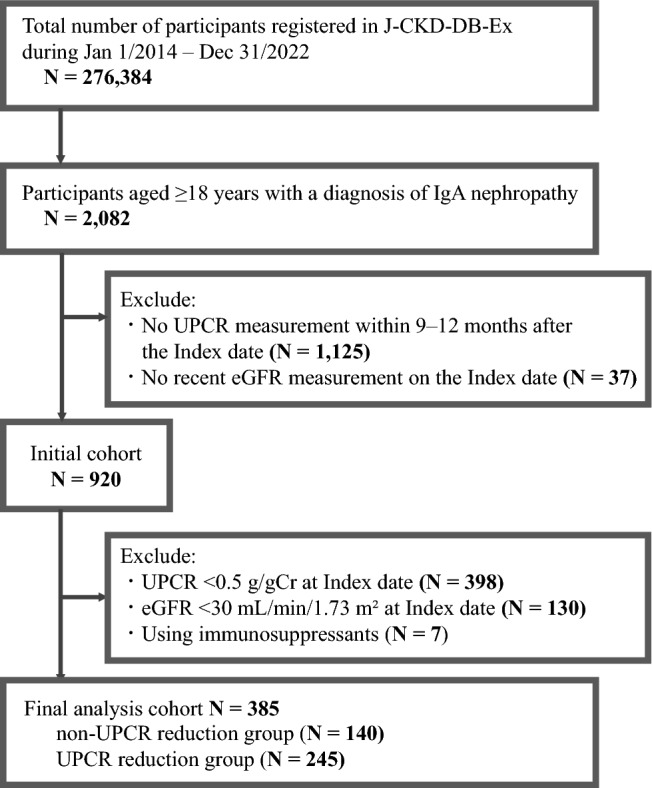
Study flowchart. Abbreviations: UPCR, urine protein/creatinine ratio; eGFR, estimated glomerular filtration rate

The database comprised 276,384 participants, of whom 2,082 were aged ≥ 18 years and had a confirmed diagnosis of IgAN. After excluding 1,125 participants without UPCR measurements within 9–12 month post-index date and 37 without recent eGFR data at the index date, 920 participants remained. Further exclusions included 398 with UPCR < 0.5 g/gCr, 130 with eGFR < 30 mL/min/1.73 m^2^, and 7 using immunosuppressants, respectively. This resulted in a final cohort of 385 participants, divided into two groups: 140 in the non-UPCR reduction group and 245 in the UPCR reduction group (Fig. 1). Baseline characteristics are presented in Table 1.

**Table 1:** Baseline characteristics and outcome events

Characteristic	Overall *N* = 385^*^	non-UPCR reduction group *N* = 140^*^	UPCR reduction group *N* = 245*	*p* value^**^
Age (years)	46.7 (14.5)	48.7 (13.1)	45.6 (15.2)	0.033
Sex				0.073
Female	205 (53%)	83 (59%)	122 (50%)	
Male	180 (47%)	57 (41%)	123 (50%)	
eGFR (mL/min/1.73m^2^)	64.7 (23.9)	59.5 (21.1)	67.6 (24.9)	0.002
CKD stage				0.13
Stages 1–2	193 (50%)	63 (45%)	130 (53%)	
Stage 3a	101 (26%)	36 (26%)	65 (27%)	
Stage 3b	91 (24%)	41 (29%)	50 (20%)	
UPCR (g/gCr)	1.8 (1.6)	1.5 (1.1)	2.0 (1.8)	< 0.001
RAS inhibitor user	29 (7.5%)	12 (8.6%)	17 (6.9%)	0.6
SGLT2 inhibitor user	0 (0%)	0 (0%)	0 (0%)	
Steroid user	5 (1.3%)	0 (0%)	5 (2.0%)	0.2
Endpoint: 40% eGFR Decline	107 (28%)	54 (39%)	53 (22%)	< 0.001
Endpoint: eGFR < 15 mL/min/1.73m^2^	34 (8.8%)	25 (18%)	9 (3.7%)	< 0.001
Composite event	107 (28%)	54 (39%)	53 (22%)	< 0.001
Average observation period (days)	2,040.3 (880.8)	2,073.2 (893.7)	2,021.4 (874.6)	0.4

Data are expressed as *N* (%) for categorical values and mean (standard deviation) for continuous values

UPCR, urine protein/creatinine ratio; eGFR, estimated glomerular filtration rate; RAS, renin–angiotensin system; SGLT2, sodium/glucose cotransporter 2

^*^Mean (SD); *n* (%)

^**^Wilcoxon rank sum test; Pearson's chi-squared test; Fisher's exact test

The overall mean age was 46.7 ± 14.5 years, mean eGFR was 64.7 ± 23.9 mL/min/1.73 m^2^, and mean UPCR was 1.8 ± 1.6 g/gCr. Overall, the mean observation period was 2040.3 ± 880.8 days, with 2073.2 ± 893.7 days and 2021.4 ± 874.6 days in the non-UPCR and UPCR reduction groups, respectively. The number of renal composite events during this period occurred in 54 (39%) and 53 participants (22%) in the non-UPCR and UPCR reduction groups, respectively. In particular, new-onset eGFR < 15 mL/min/1.73 m^2^ occurred in 54 participants (39%) in the non-UPCR reduction group and 53 participants (22%) in the UPCR reduction group, while a 40% decline in eGFR occurred in 25 (18%) and 9 participants (3.7%), respectively.

### UPCR reduction and ESKD risk

**Fig. 2:**
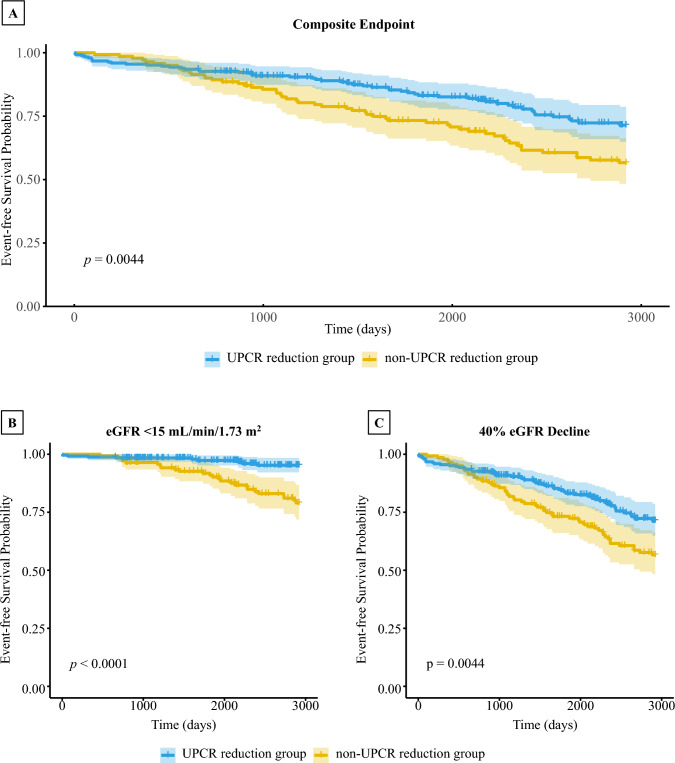
Kaplan–Meier survival curves for renal composite outcome according to 30% UPCR reduction. Kaplan–Meier curves with log-rank test were used to compare renal outcomes between participants with and without a 30% reduction in UPCR. Panels show renal composite events (**A**), new onset of eGFR < 15 mL/min/1.73 m^2^ (**B**), and 40% eGFR decline (**C**). The blue line represents the Kaplan–Meier survival curve for the UPCR reduction group with the shaded blue area indicating the 95% confidence interval (CI), while the yellow line represents the survival curve for the non-UPCR reduction group with the shaded yellow area indicating the 95% CI. Mean follow-up was 2040.3 ± 880.8 days. Abbreviations: UPCR, urine protein/creatinine ratio; eGFR, estimated glomerular filtration rate

The mean interval between the index date and the second UPCR measurement was 9.9 months. We investigated the association between the presence or absence of a 30% UPCR reduction and the occurrence of renal composite event, new onset of eGFR < 15 mL/min/1.73 m^2^, and 40% eGFR decline using the Kaplan–Meier curves with log-rank test for group comparisons. The cumulative incidence of renal composite event was significantly lower in the UPCR reduction group than that in the non-UPCR reduction group (Fig. 2a). Similar results were observed for new onset of eGFR < 15 mL/min/1.73 m^2^ (Fig. 2b) and 40% eGFR decline (Fig. 2c). The Schoenfeld residuals test did not reveal any evidence of violation of the proportional hazards assumption (Supplementary Fig. 1).

**Fig. 3:**
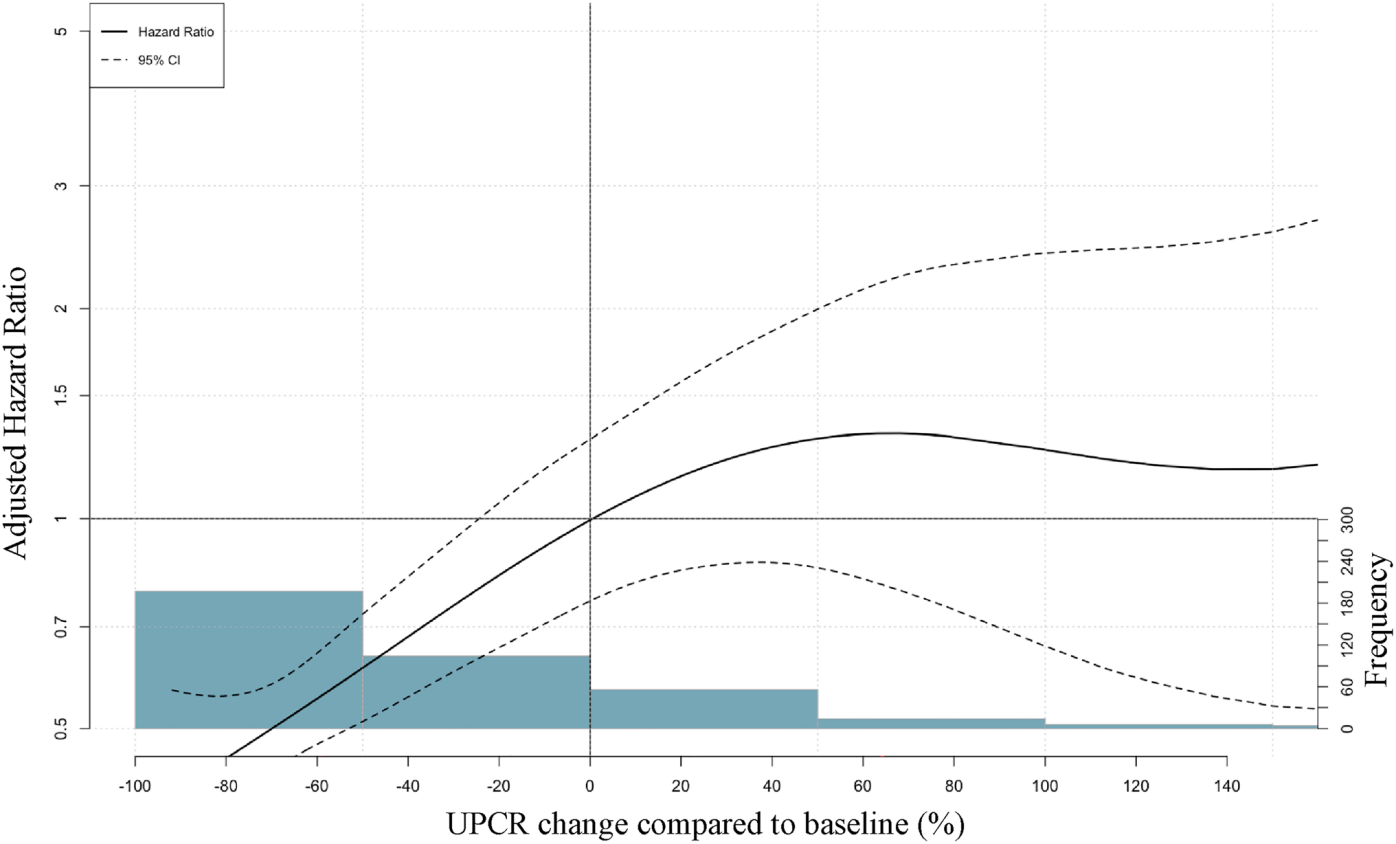
Restricted cubic spline analysis of the association between UPCR change and renal composite events. Restricted cubic spline analysis shows the association between UPCR change and the risk of renal composite events. Multivariable models were adjusted for age, sex, baseline eGFR, baseline UPCR, and renin–angiotensin system inhibitor use. The solid line represents the estimated hazard ratio, and the dashed lines indicate the 95% confidence interval. Reference for the spline was 0% UPCR change; shaded bars depict the sample distribution of percent change. Abbreviations: UPCR, urine protein/creatinine ratio; eGFR, estimated glomerular filtration rate

Subsequently, the risk of renal composite event was estimated by multivariate analysis using a Cox proportional hazards model. The UPCR reduction group had a significantly lower adjusted Hazard Ratio (aHR) for renal composite events than the non-UPCR reduction group at 0.53 (95% CI 0.35–0.79) (Table 2a). Similarly, the aHR was 0.16 (95% CI 0.07–0.39) for new onset of eGFR < 15 mL/min/1.73 m^2^, and 0.45 (95% CI 0.30–0.68) for 40% eGFR decline, indicating a significant reduction in the risk of renal failure event in the UPCR reduction group (Table 2b). In restricted cubic spline models, the hazard decreased progressively with greater UPCR reductions, with no clear inflection point or sharp threshold (Fig. 3).

**Table 2:** Adjusted hazard ratios for renal outcomes according to UPCR reduction

	Unadjusted model			Adjusted model		
Characteristic	HR	95% CI	*p* value	aHR	95% CI	*p* value
(a)						
UPCR ≥ 30% reduction	0.58	0.40, 0.85	0.005	0.53	0.35, 0.79	0.002
Age				0.99	0.98, 1.01	0.5
Sex (Male)				1.29	0.88, 1.90	0.2
eGFR				0.98	0.97, 0.99	< 0.001
UPCR				1.27	1.17, 1.38	< 0.001
RAS Inhibitor use				1.24	0.62, 2.48	0.6

	eGFR < 15 mL/min/1.73m^2^			40% eGFR Decline		
Characteristic	aHR	95% CI	*p* value	aHR	95% CI	*p* value
(b)						
UPCR ≥ 30% reduction	0.16	0.07, 0.39	< 0.001	0.53	0.35, 0.79	0.002
Age	0.98	0.95, 1.01	0.13	0.99	0.98, 1.01	0.5
Sex (Male)	1.11	0.55, 2.25	0.8	1.29	0.88, 1.90	0.2
eGFR	0.94	0.92, 0.97	< 0.001	0.98	0.97, 0.99	< 0.001
UPCR	1.24	1.08, 1.43	0.002	1.27	1.17, 1.38	< 0.001
RAS inhibitor use	1.06	0.31, 3.56	> 0.9	1.24	0.62, 2.48	0.6

Renal outcomes were analyzed as renal composite events (a) and new onset of eGFR < 15 mL/min/1.73 m^2^ or 40% eGFR decline (b) compared between the UPCR and non-UPCR reduction groups. The risk of renal composite events was estimated by multivariate analysis using a Cox proportional hazards model, adjusted for baseline covariates, including age, sex, baseline eGFR, baseline UPCR, and RAS inhibitor use

HR, Hazard Ratio; aHR, adjusted Hazard Ratio; CI, Confidence Interval; UPCR, urine protein/creatinine ratio; eGFR, estimated glomerular filtration rate; RAS, renin–angiotensin system

### Subgroup analysis

**Fig. 4:**
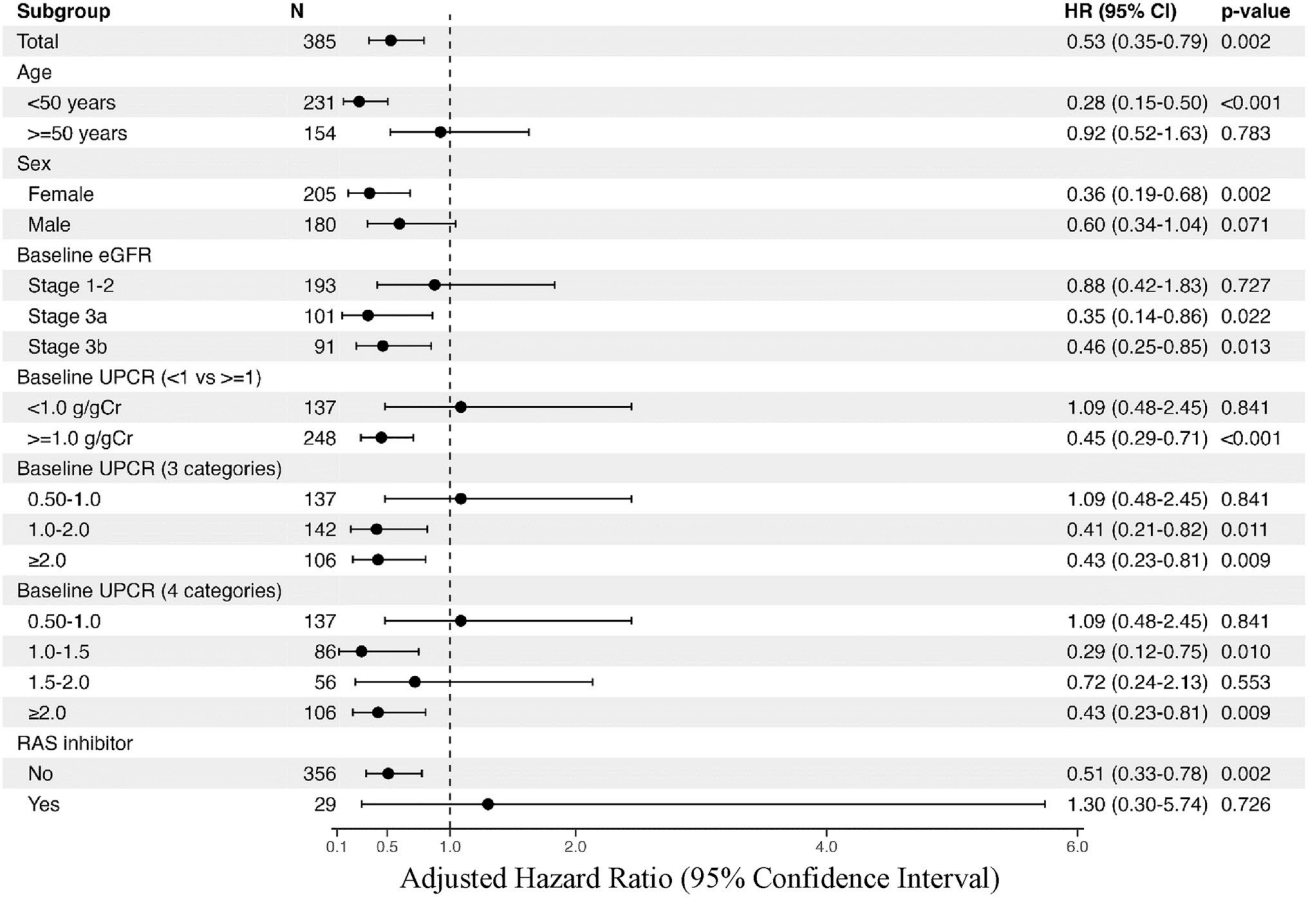
Adjusted hazard ratios for renal composite events according to UPCR reduction in each subgroup. The adjusted hazard ratios for renal composite events in the UPCR reduction group compared to the non-UPCR reduction group are shown for each subgroup. Multivariable models were adjusted for age, sex, baseline eGFR, baseline UPCR, and RAS inhibitor use. Abbreviations: UPCR, urine protein/creatinine ratio; eGFR, estimated glomerular filtration rate; RAS, renin–angiotensin system

The aHRs for renal composite events in the UPCR reduction group compared to the non-UPCR reduction group are shown for each subgroup (Fig. 4). A substantial reduction in renal failure risk was observed in the group < 50 years (aHR 0.28, 95% CI 0.15–0.50), whereas no significant risk reduction was found in the group aged ≥ 50 years. Greater risk reduction was associated with more advanced kidney disease, with aHRs of 0.46 (95% CI 0.25–0.85) for CKD stage G3b (30 ≤ eGFR < 45 mL/min/1.73 m^2^) and 0.35 (95% CI 0.14–0.86) for CKD stage G3a (45 ≤ eGFR < 60 mL/min/1.73 m^2^), compared to an aHR of 0.88 (95% CI 0.42–1.83) for CKD stages G1–2 (eGFR ≥ 60 mL/min/1.73 m^2^). Strong risk reduction was observed for baseline proteinuria in the group with > 1 g/gCr (aHR 0.45, 95% CI 0.29–0.71); however, no significant risk reduction was found in the group with < 1 g/gCr. Regarding RAS inhibitor use, few cases were receiving prescriptions at baseline, and the aHR for non-RAS inhibitor users was very close to that of the overall cohort.

### UPCR reduction and eGFR slope

We examined the relationship between UPCR reduction and the eGFR slope. The eGFR slope was calculated using values from the index date to the end of the observation period (Table 3a). As a sensitivity analysis, the slope was also calculated using eGFR values from the index date for up to 3 years (Table 3b). In the non-UPCR reduction group, the mean eGFR slope showed a decline of -3.4 mL/min/1.73 m^2^/year, whereas the UPCR reduction group exhibited a slower decline at -1.9 mL/min/1.73 m^2^/year, resulting in a difference of 1.5 mL/min/1.73 m^2^/year. The respective declines for the 3-year period were -3.8 and -1.8 mL/min/1.73 m^2^/year, yielding a difference of 2.0 mL/min/1.73 m^2^/year. Therefore, we assessed whether a greater percentage decrease in UPCR was associated with a more favorable (less steep) eGFR slope to further investigate the impact of UPCR reduction on kidney function. We evaluated the relationship between the rate of change in UPCR, treated as a continuous variable, and the eGFR slope. The results indicated that a 1% decrease in UPCR corresponded to a 0.01 mL/min/1.73 m^2^/year slower decline in eGFR (Table 4). In addition, the UPCR change rate was categorized into seven groups: “ ≥ 40% increase,” “ ≥ 20% increase,” “within ± 20%,” “ ≥ 20% decrease,” “ ≥ 40% decrease,” “ ≥ 60% decrease,” and “ ≥ 80% decrease.” [14] Inter-group differences in eGFR slope were calculated using the “within ± 20%” group as the reference. The results revealed that as UPCR decreased, the eGFR slope became less steep, and increased UPCR was associated with a steeper eGFR decline (Supplementary Fig. 2).

**Table 3:** Association between UPCR reduction and eGFR slope

Characteristic	Beta (mL/min/1.73 m^2^/year)	95% CI	Reference
(a) eGFR slope estimated from all available follow-up data			
Time (years)	− 3.4	− 3.9, − 2.9	–
UPCR ≥ 30% reduction	5.5	1.3, 9.7	Non-UPCR reduction
Age (per year)	− 0.84	− 0.97, − 0.70	–
Sex (Male)	− 5.9	− 9.8, − 2.0	Female
UPCR (per unit)	− 1.8	− 3.0, − 0.56	–
RAS inhibitor use	− 8.0	− 15, − 0.68	Not used
Interaction: Time × UPCR reduction	1.5	− 0.87, 2.1	–

Characteristic	Beta (mL/min/1.73 m^2^/year)	95% CI	Reference
(b) eGFR slope estimated from the first 3 years of follow-up data			
Time (years)	− 3.8	− 4.6, − 3.1	–
UPCR ≥ 30% reduction	5.0	0.71, 9.2	Non-UPCR reduction
Age (per year)	− 0.84	− 0.98, − 0.70	–
Sex (Male)	− 5.9	− 9.8, − 1.9	Female
UPCR (per unit)	− 1.8	− 3.0, − 0.53	–
RAS inhibitor use	− 8.1	− 15, − 0.67	Not used
Interaction: Time × UPCR reduction	2.0	1.1, 3.0	–

Annual eGFR decline was assessed using linear mixed-effects models with random intercepts and slopes, utilizing all available eGFR measurements during follow-up. Analyses were conducted for (a) the entire follow-up period and (b) restricted to the first 3 years as a sensitivity analysis. Differences in eGFR slopes between the UPCR and non-UPCR reduction groups were evaluated after adjusting for baseline covariates, including age, sex, baseline eGFR, baseline UPCR, and renin–angiotensin system inhibitor use. The non-UPCR reduction group was used as the reference. The interaction term represents the difference in eGFR slope between UPCR reduction groups, and a positive beta coefficient indicates a slower eGFR decline in the UPCR reduction group

CI, Confidence Interval; UPCR, urine protein/creatinine ratio; eGFR, estimated glomerular filtration rate; RAS, renin–angiotensin system

**Table 4:** Association between continuous UPCR change and eGFR slope

	Univariate			Multivariable		
Characteristic	Beta	95% CI	*p* value	Beta	95% CI	*p* value
(Intercept)	− 2.9	− 3.2, − 2.6	< 0.001	− 1.3	− 3.0, 0.32	0.11
UPCR change (per percent)	− 0.01	− 0.02, − 0.01	< 0.001	− 0.01	− 0.02, − 0.01	< 0.001
Age				0.01	− 0.02, 0.03	0.6
Sex (Male)				− 0.21	− 0.73, 0.31	0.4
eGFR (per 1 mL/min/1.73m^2^)				− 0.02	− 0.04, − 0.01	< 0.001
UPCR (per 1 g/gCr)				− 0.19	− 0.35, − 0.03	0.022
RAS inhibitor use				− 0.06	− 1.0, 0.90	0.9

The eGFR slope was assessed using linear mixed-effects models with random intercepts and slopes, utilizing all available eGFR measurements during the entire follow-up period. Univariate and multivariable analyses were performed. Multivariable models were adjusted for baseline covariates, including age, sex, baseline eGFR, baseline UPCR, and renin–angiotensin system inhibitor use. Differences in eGFR slopes between the UPCR and non-UPCR reduction groups were evaluated. The non-UPCR reduction group was used as the reference

CI, Confidence Interval; UPCR, urine protein/creatinine ratio; eGFR, estimated glomerular filtration rate; RAS, renin–angiotensin system

### *Achievement of UPCR* < *1 g/Cre and the risk of ESKD*

Based on the subgroup analysis results according to baseline proteinuria levels, participants with baseline UPCR ≥ 1 g/gCr were stratified into two groups: those who achieved UPCR < 1 g/gCr at 9–12 months and those who did not. The subsequent risk of renal composite events was estimated. Participants who achieved UPCR < 1.0 g/gCr showed a significantly better renal prognosis, with an adjusted HR of 0.39 (95% CI, 0.26–0.59) (Supplementary Table).

## Discussion

In this study, we examined whether proteinuria reduction, an endpoint supported predominantly by research from the United States, as a surrogate for ESKD in IgAN, can be extrapolated to the Japanese population with IgAN using real-world data (J-CKD-DB-Ex). Our findings demonstrate that urinary protein reduction is associated with subsequent renal events and changes in eGFR slope among Japanese patients with IgAN, supporting the potential generalizability of this surrogate endpoint.

A 30% reduction in proteinuria from baseline at 9–12 months was associated with a significant reduction in the risk of eGFR 40% decline, new onset of eGFR < 15 mL/min/1.73 m^2^, and a composite endpoint of both, compared to participants without such a reduction. We adopted a composite endpoint of new onset of eGFR < 15 mL/min/1.73 m^2^ and new onset of eGFR 40% decline as our primary endpoint, because the database lacked information on true hard endpoints, such as dialysis initiation and kidney transplantation. Moreover, a 40% decline in eGFR is well-established as a robust surrogate endpoint for renal failure [15] and has been used as a renal composite endpoint in several clinical trials. Although the appropriateness of adopting new onset of eGFR < 15 mL/min/1.73 m^2^, i.e., progression to CKD stage G5, as a surrogate endpoint has not been sufficiently validated, we believe that treating these two endpoints as a composite makes it more robust and the findings from this study support the generalizability of early proteinuria reduction as a surrogate endpoint in the Japanese IgAN population. However, further interventional trials or validation through multiple cohort studies—including those involving Japanese patients with IgAN—are necessary to confirm whether proteinuria reduction is indeed associated with a decreased risk of future renal failure events.

Subgroup analyses revealed some heterogeneity, likely because of small sample sizes or event counts in certain groups. Notably, significant risk reductions were observed among participants under 50 years of age and those with UPCR > 1 g/gCr. Previous studies investigating the age distribution at diagnosis of IgAN in Japan have shown a bimodal peak in the teenage and early 40 s age groups [16], with registry-based data indicating a broad diagnostic age range from 10 to 50 s, peaking around 30–39 years [17]. The observed risk reduction in patients under 50 years suggests that urinary protein decline may serve as a marker of a favorable clinical course and potentially reduced future renal progression in newly diagnosed patients. Furthermore, previous observational studies have demonstrated higher incidences of ESKD in patients with proteinuria > 1 g/day. For instance, one study reported a significantly higher rate of ESKD when urinary protein > 1 g/day [18], whereas another found a 46.5-fold increased ESKD risk in individuals with proteinuria ≥ 1.0 g/day compared to those with < 0.5 g/day [19]. Similarly, this study showed that proteinuria reduction was closely associated with renal failure risk reduction in participants with proteinuria > 1 g/gCr, in whom poor renal prognosis was expected.

In this study, we used a 30% reduction in urinary protein as an explanatory factor for risk estimation, consistent with the sample size calculation of the PROTECT trial [6], which assumed a true relative treatment effect of over 30% for proteinuria reduction. In addition, early proteinuria reduction was a key criterion for accelerated approval in clinical trials of budesonide [5], sparsentan [6], iptacopan [7], and atrasentan [8], where the observed reduction rates at 9 months were 27%, 41%, 38%, and 36%, respectively. Notably, all of these agents demonstrated preservation of renal function at the final follow-up, supporting early proteinuria reduction as a strong surrogate endpoint (the final renal outcomes for iptacopan and atrasentan are still under evaluation at the time of writing). Although the optimal cutoff for proteinuria reduction remains undefined, a 30% decrease appears justifiable based on these clinical trial results. Moreover, a greater magnitude of proteinuria reduction has been linearly associated with a lower risk of true clinical endpoints [20]. Consistently, our data showed that participants with ≥ 30% reduction in urinary protein had a significantly lower long-term risk of renal failure. In addition, larger reductions correlated with greater improvements in the eGFR slope and lower HRs for renal composite events when considering the degree of change in proteinuria as a continuous or categorical variable. In particular, the inter-group differences fell within the range (0.5–1.0 mL/min/1.73 m^2^/year) considered effective for eGFR slope as a surrogate endpoint in early CKD [21–23]. These findings support the use of early proteinuria reduction as a valid surrogate endpoint in Japanese IgAN populations.

Nonetheless, this study has some limitations. First, this study is an observational analysis based on a database derived from electronic health records, which could not account for unmeasured confounders, such as blood pressure, smoking status, and physical activity—factors potentially significantly affecting renal prognosis. In particular, the influence of blood pressure on urinary protein is thought to be substantial but could not be adjusted for herein. Second, data on treatments such as tonsillectomy with corticosteroid therapy were not available; given that these are common therapies for IgAN in Japan, their absence may introduce confounding bias. Third, information on mortality was not included, preventing analysis of all-cause mortality as an outcome. Deaths serve as competing risks for renal events, and their omission may lead to an underestimation of renal event risks. Fourth, multiple sources of selection bias are present. For instance, the J-CKD-DB-Ex registry primarily covers university hospitals; thus, it may include a higher proportion of severe IgAN cases. Finally, this study did not evaluate changes in urinary protein following specific treatment interventions; thus, the observed associations reflect a composite of various clinical courses and management strategies. Based on these findings, it can be suggested that achieving at least a 30% reduction in proteinuria within approximately 9 months from a specific outpatient visit would be desirable for Japanese patients with IgA nephropathy. In contrast, clinical trials of new therapeutic agents establish the specific intervention initiation as baseline to evaluate early proteinuria reduction effects, making direct comparisons with our findings less appropriate. Consequently, further validation through prospective cohort or intervention studies is necessary for more definitive conclusions.

## Conclusion

Using the Japanese CKD database J-CKD-DB-Ex, we examined whether early urinary protein reduction, a surrogate endpoint widely adopted in international clinical trials, is appropriate for Japanese patients with IgAN. A ≥ 30% reduction in urinary protein at 9–12 months was associated with decreased subsequent renal failure risk and attenuation of eGFR decline. These findings suggest that early proteinuria reduction is a valid surrogate endpoint for renal prognosis in Japanese IgAN populations.

## Supplementary Information

Below is the link to the electronic supplementary material.

Supplementary file1 (PDF 494 KB)

## Data Availability

The data sets were generated at Kawasaki Medical School. Data supporting the findings are available from the corresponding author upon reasonable request.
