# Supplementary material for: Proteinuria reduction as a surrogate endpoint for clinical study of IgA nephropathy in Japanese patients: data from the J-CKD-DB-Ex
Source: Clin Exp Nephrol. 2025 Nov 18;30(2):309–19. doi: 10.1007/s10157-025-02788-4 (PMC12886275; doi:10.1007/s10157-025-02788-4)
Supplement: Supplementary file 1 — Supplementary file1 (PDF 494 KB) [file 10157_2025_2788_MOESM1_ESM.pdf]

Supplementary materials

Proteinuria reduction as a surrogate endpoint for clinical study of IgA nephropathy in Japanese patients: data from the J-CKD-DB-Ex

Clinical and Experimental Nephrology

Naoki Kashihara 1, Seiji Itano 2, Takaya Nakashima 3, Tadahiro Goto 4, Keisuke Yoshihara 5, Shunsuke Eguchi 5, Kazuma Iekushi 5, Yoshitaka Isaka 6, Hajime Nagasu 2, and J-CKD-DB collaborators.

Affiliations:

1. Geriatric Medical Center, Kawasaki Medical School, Okayama, Japan

2. Department of Nephrology and Hypertension, Kawasaki Medical School, Kurashiki, Okayama, Japan

3. Department of Anesthesiology and Intensive Care Medicine, Nagasaki University Graduate School of Biomedical Sciences, Nagasaki, Japan

4. Department of Health Data Science, Graduate School of Data Science, Yokohama City University, Kanagawa, Japan

5. Medical Affairs, Novartis Pharma K.K., Tokyo, Japan

6. Department of Nephrology, Osaka University Graduate School of Medicine, Osaka, Japan.

Corresponding Author:

Seiji Itano, MD, PhD

Department of Nephrology and Hypertension

Kawasaki Medical School

577 Matsushima, Kurashiki, Okayama, Japan, 701-0192

Mail: s.itano@med.kawasaki-m.ac.jp

Supplementary Method

Calculation of eGFR

The eGFR values are calculated from serum creatinine levels using the Japanese equation for estimating GFR [24].

24. Matsuo S, Imai E, Horio M, Yasuda Y, Tomita K, Nitta K, et al. Revised equations for estimated GFR from serum creatinine in Japan. Am J Kidney Dis. 2009; 53:982–92. doi: 10.1053/j.ajkd.2008.12.034.

Statistical Analysis

Baseline comparisons

Continuous variables are expressed as mean ± standard deviation and compared using the Wilcoxon rank-sum test. Categorical variables are presented as numbers (percentages) and were compared using the Pearson's chi-squared test or Fisher exact test, as appropriate.

Time-to-event analyses

Kaplan–Meier curves with log-rank tests were used for group comparison. Cox proportional hazards models were used to estimate the hazard ratios (HRs) with 95% confidence intervals (CIs). Both unadjusted and adjusted models were used. The proportional hazards assumption was tested using the Schoenfeld residuals.

Restricted cubic spline analyses

Multivariable Cox models with restricted cubic splines (reference = 0% UPCR change; three knots at the 10th, 50th, and 90th percentiles) were used to assess the dose–response between percent UPCR change and renal outcomes, with interpretation based on the spline shape and 95% confidence intervals.

eGFR slope analyses

Annual eGFR decline was assessed using linear mixed-effects models with random intercepts and slopes, using all available eGFR data. Analyses were performed for the entire follow-up period and restricted to the first 3 years as a sensitivity analysis. Group differences in the slopes were evaluated after adjusting for baseline covariates.

Subgroup and sensitivity analyses

Prespecified subgroup analyses were stratified by age (<50 vs ≥50 years), sex, CKD stage, baseline UPCR categories, and renin-angiotensin system (RAS) inhibitor use. Sensitivity analyses were performed to evaluate the individual components of the primary endpoint.

Software and significance

All analyses were conducted using R version 4.3.2 (R Foundation for Statistical Computing, Vienna, Austria). A two-sided $P < 0.05$ was considered to be statistically significant.

Covariates

The following baseline covariates were included: sex, age, eGFR, UPCR, and prescription of RAS inhibitors and corticosteroids. A medication prescription was defined as present if a prescription was recorded within 90 days prior to the index date, up to and including the index date. A complete case analysis, including only participants with complete data for all specified covariates, was performed.

Supplementary Table

Adjusted hazard ratios for renal outcomes based on achievement of UPCR <1 g/gCr

Characteristic	aHR	95% CI	p-value
UPCR < 1.0 g/gCr at 9–12 months (Ref: UPCR ≧ 1.0 g/gCr at 9–12 months)	0.39	0.26, 0.59	<0.001
Age	1.00	0.98, 1.01	0.55
Sex (Male)	1.39	0.94, 2.04	0.10
eGFR	0.98	0.97, 0.99	<0.001
UPCR	1.18	1.09, 1.28	<0.001
RAS Inhibitor use	1.41	0.70, 2.83	0.34

Comparison of renal composite event risk between participants with baseline proteinuria >1 g/day who achieved proteinuria <1 g/day at 9–12 months and those who did not. The risk of renal composite events was estimated by multivariate analysis using a Cox proportional hazards model, adjusted for baseline covariates including age, sex, baseline eGFR, baseline UPCR, and RAS inhibitor use.

Abbreviations: HR, Hazard Ratio; aHR, adjusted Hazard Ratio; CI, Confidence Interval; UPCR, urine protein/creatinine ratio; eGFR, estimated glomerular filtration rate; RAS, renin–angiotensin system.

Supplement figure1

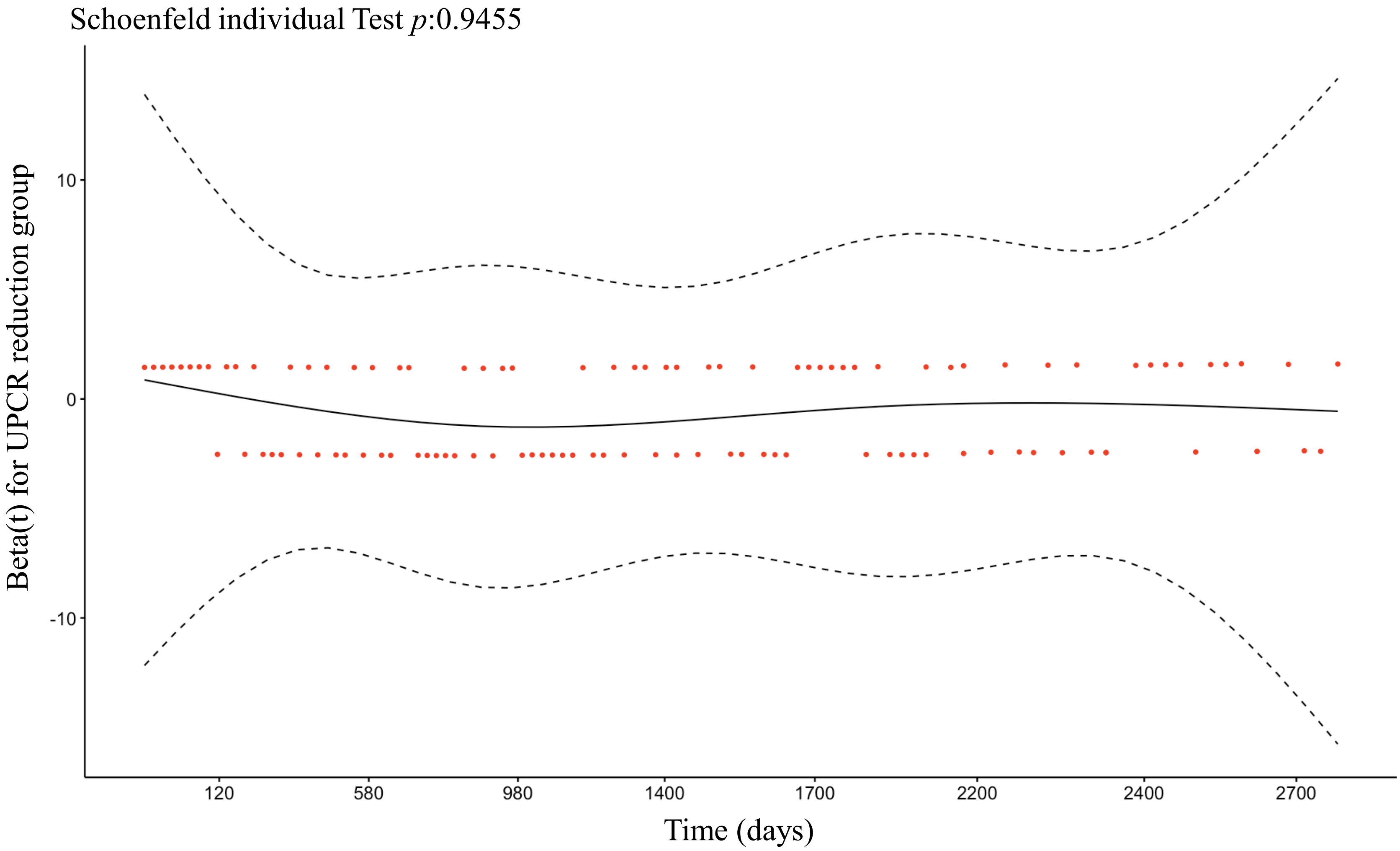

Supplementary Figure 1. Schoenfeld residual plot for the UPCR reduction group

Schoenfeld residuals for the UPCR reduction group were plotted against time. The smoothed curve appeared approximately horizontal, indicating no violation of the proportional hazards assumption. The Schoenfeld individual test showed no evidence of violation (p = 0.9455). Abbreviations: UPCR, urine protein/creatinine ratio.

Supplement figure2

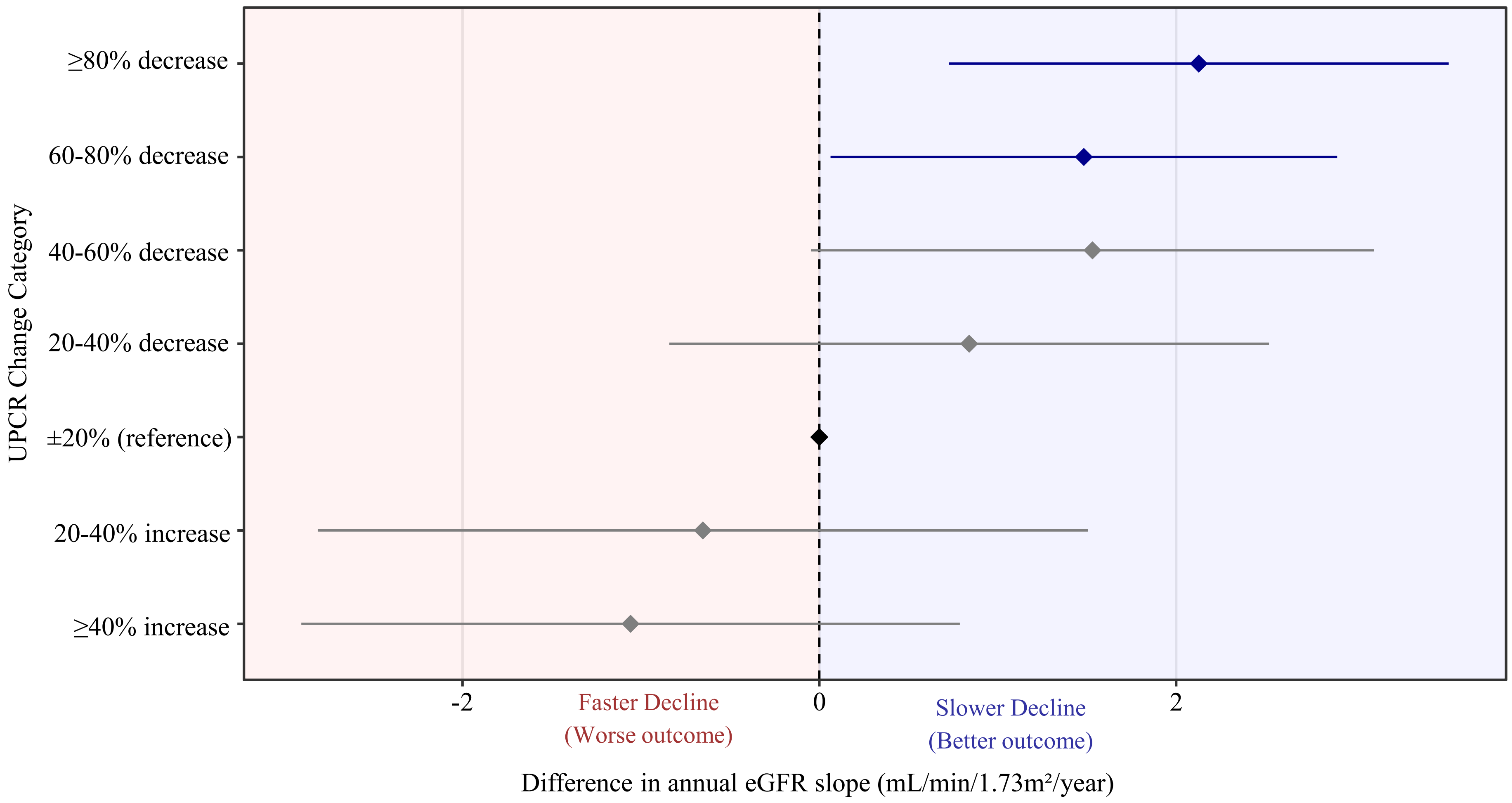

Supplementary Figure 2. Association between categorized UPCR change and eGFR slope

The UPCR change rate was categorized into seven groups: "≥40% increase," "≥20% increase," "within ±20%," "≥20% decrease," "≥40% decrease," "≥60% decrease," and "≥80% decrease." Using the "within ±20%" group as the reference, inter-group differences in eGFR slope were evaluated. Abbreviations: UPCR, urine protein/creatinine ratio; eGFR, estimated glomerular filtration rate.
